# Vertical augmentation of the atrofic posterior mandibular ridges with onlay grafts: Intraoral blocks vs. guided bone regeneration. Systematic review

**DOI:** 10.4317/jced.60294

**Published:** 2023-05-01

**Authors:** Louise Robert, Amparo Aloy-Prósper, Santiago Arias-Herrera

**Affiliations:** 1 Universidad Europea de Valencia. Faculty of Health Sciences. Department of Dentistry; 2 Assistant Professor of Oral Surgery, Stomatology Department, Faculty of Medicine and Dentistry, University of Valencia, Valencia, Spain

## Abstract

**Background:**

The high resorption rate of intra-oral Onlay block grafts, coupled with morbidity and limited bone availability, means that the use of guided bone regeneration (GBR) may be preferable for vertical augmentation of mandibular atrophic posterior sectors. Aims: To evaluate the bone gain and surface resorption of the intraoral Onlay block graft compared to the GBR; as well as to study postoperative complications, survival and success rates of dental implant, and peri-implant marginal bone loss.

**Material and Methods:**

An electronic search was performed in the PubMed, Scopus, and Web Of Science databases on bone augmentation with intraoral autologous onlay block graft or GBR until December 2021.

**Results:**

Of 214 potentially eligible papers, 11 complied with the inclusion criteria: 5 studies on block graft technique, 5 on GBR technique and 1 was a comparison of both treatment groups. In the block graft group, the mean vertical bone gain was 4.05mm with a mean resorption of 0.84mm (17.70%); the complication rate was 20%; the survival and success rates were 100% and 92.23% respectively and the mean peri-implant bone loss was 0.22mm at 12 months. For the GBR group, the mean bone gain was 4.7mm with a mean resorption of 0.33mm (15.08%); the complication rate was 11.6%; the survival rate was 100% and the mean peri-implant bone loss was 0.95mm at 12 months.

**Conclusions:**

Despite the limitations, the GBR technique seems to achieve greater bone gain with less superficial resorption as well as fewer complications, but it presents a greater peri-implant loss at 12 months.

** Key words:**Onlay block graft, Guided Bone Regeneration, intraoral bone, augmentation procedure.

## Introduction

Vertical bone augmentation of the alveolar ridges is considered one of the great challenges in the field of implantology, especially when it comes to the posterior sectors of the atrophic mandible, since the presence of the inferior alveolar dental nerve represents a significant anatomical limitation (1). Bone regeneration by Onlay intraoral block grafts or by Guided Bone Regeneration (GBR) technique represents an appropriate therapeutic option for the reconstruction of the alveolar process (2). The superficial resorption, the limited bone availability and the morbidity associated with autogenous block grafts, together with the good osteoconductive properties of particulate bone substitutes, make the use of Guided Bone Regeneration technique preferable in an attempt to overcome limitations of this type of graft (3). There are several published systematic reviews on vertical augmentation with Guided Bone Regeneration and block grafts (4-6); however, in these reviews, in addition to intraoral autologous grafts, they included other types of origin (extraoral autologous; xenografts; allografts and synthetic) and did not differentiate the results from each other (4-6); while others included deferred implants as well as simultaneous implants without separating the data between the groups (4,5). Urban *et al*. (4) and Hameed *et al*. (5) reviewed both the vertical bone augmentation in maxilla and mandible, anterior and posterior, but did not separate the data. While the review published by Camps-Font *et al*. (6) although it focused on the posterior vertical augmentation of the atrophic jaw; it included as a main variable the implant survival and success. However, the present systematic review has focused on evaluating bone gain achieved with the vertical augmentation procedure.

The aim of the present systematic review was to systematically review the following question: In patients with vertical atrophy of the mandibular posterior ridges who had undergone an Onlay-type bone augmentation procedure, does Guided Bone Regeneration with particulate grafting result in greater bone gain and less superficial bone resorption than intraoral autogenous block grafts? This was done by firstly assessing the bone gain and resorption rate measurements, and secondly evaluating complications related to the augmentation procedure, implant survival, implant success, and radiographic peri-implant marginal bone loss.

## Material and Methods

This systematic review complies with the PRISMA statement (Preferred Reporting Items for Systematic reviews and Meta-Analyses) (7).

- Focus question:

The focus question was established according to the PICO structured question:

P (population): Patients with vertical atrophy of the mandibular posterior ridges who had undergone an Onlay-type bone augmentation procedure.

I (intervention): Guided Bone Regeneration (particulate bone with membrane-barrier).

C (comparison): Intraoral Onlay block bone graft.

O (outcomes):

o O1: Bone gain and resorption rate.

o O2: Postoperative complications related to the bone augmentation procedure.

o O3: Survival and success implant rates, and peri-implant marginal bone loss.

- Eligibility criteria:

The inclusion criteria were:

• Study design: randomized clinical trials, prospective and retrospective cohort studies, case series, studies on humans, ≥5 patients; publication in English or Spanish or French, up to December 2021.

• Patient: patients with vertical bone atrophy of the mandibular posterior ridges treated with bone grafts.

• Intervention: Onlay-type bone augmentation with block grafts obtained from intraoral regions or through a Guided Bone Regeneration procedure, with a minimum follow-up of 4 months after augmentation procedure.

• Outcomes: Studies that include data related to the bone gain and/or resorption rate as main variables. And as secondary variables: postoperative complications related to the augmentation procedure, survival and success rates of dental implants and peri-implant marginal bone loss.

The exclusion criteria were reviews, case report, letters or comments to the editor, expert reports, *in vitro* and animal experimental studies. Also excluded, studies in which only horizontal augmentation, alveolar bone distraction, inlay-type grafts, alveolar preservation or LeFort surgeries, GBR simultaneous to implant placement, as well as other regeneration procedures not described as included were performed.

No restrictions were imposed according to the year of publication. Authors were contacted for clarification of missing information when necessary.

- Information sources and data search:

An automatized electronic and manual literature searches were conducted in three major electronic databases (PubMed, Scopus and Web of Science) with the following keywords: “edentulous jaw”, “edentulous mandible”, “atrophied jaw”, “jaw atrophy”, “bone graft”, “bone regeneration”, “bone augmentation”, “vertical ridge augmentation”, “guided bone regeneration”, “autogenous bone”, “autologous bone”, “intraoral bone”, “intraoral onlay block”, “onlay bone graft”, “block graft”, “bone block graft”, “bone gain”, “resorption”, “complication”. Keywords were combined with a combination of the controlled terms (MeSH for Pubmed) to obtain the best search results.

The following search strategy in Pubmed was carried out: ((“Jaw, Edentulous”[Mesh] OR “Jaw, Edentulous, Partially”[Mesh] OR “Mouth, Edentulous”[Mesh] OR partial edentulous patients OR edentulous patient OR edentulous jaw OR atrophied jaw OR atrophic arch OR jaw atrophy OR bone atrophy OR deficient alveolar ridge OR bone augmentation OR bone regeneration OR alveolar ridge augmentation) AND (vertical OR vertically)) AND (((intraoral OR ramus OR symphysis OR retromolar OR chin) AND (block bone graft OR corticocancellous bone graft OR onlay bone graft)) OR (guided bone regeneration OR GBR)) AND (“dental implants”[MeSH Terms] OR dental implant) AND (bone gain OR mean bone regeneration OR resorpt*) NOT sinus NOT furcation defect NOT socket. Filters: Humans, English, French, Italian, Spanish.

To identify any eligible studies that the initial search might have missed, the search was completed with a review of the references provided in the bibliography of each study. On the other hand, a manual search of scientific articles from the following oral surgery and implantology journals was carried out: Journal of Dental Research, Journal of Dentistry, Clinical Oral Implants Research, Clinical Implant Dentistry and Related Research, Journal of Periodontology, Journal of Periodontal Research, Journal of Oral Implantology and Implant Dentistry.

- Search strategy:

A selection process was carried out in three stages. Study selection was carried out by two reviewers (LR, AAP). In the first stage, titles were screened to eliminate irrelevant publications. In the second stage, abstracts were filtered according to the type of study, type of graft, type of intervention, number of patients, and outcome variables. Studies without sufficient information or with unstructured abstracts to determine their exclusion were considered for full text evaluation. The third phase consisted of a full reading of each text using a predetermined data extraction form to confirm study eligibility upon the predetermined inclusion and exclusion criteria. Disagreements between reviewers, at each of the phases, were resolved by discussion and, when necessary, a third reviewer was consulted. The degree of agreement regarding the inclusion of potential studies was calculated by k-statistics (Cohen kappa test) for the second and third stage of selection.

- Extraction data:

The following information was extracted from the studies and arranged in Tables according to the type of procedure (block graft or ROG): authors with the year of publication, type of study, number of patients, type of atrophy (vertical or vertical-horizontal), grafting site (maxillary, mandibular, anterior, posterior), number of grafts, number of implants, donor site of the graft, type of particulate graft (autogenous, allograft, xenograft, mixture and, if so, the ratio of the mixture), total bone gain (mm), superficial resorption of the graft before placing the implants (mm and/or %), follow-up (months), use of a particulate biomaterial with the block (yes, no, and if so, the type of material used), use of membrane (yes, no, and if so, the type of material used), graft healing time (months), postoperative complications related to the bone augmentation procedure (number, type of complication and its evolution or treatment), implant placement time (deferred, simultaneous), implant survival rates (%), implant success rate (% and criteria used), mean marginal bone loss (mm).

- Quality and risk of bias assessment:

Two reviewers (LR, AAP) independently evaluated the methodological quality of the included studies. Cochrane 5.1.0 (http://handbook.cochrane.org) guidelines were used to evaluate the quality of randomized controlled clinical trials; publications were considered “low risk of bias” when they m*et al*l criteria, “high risk of bias” when one or more criteria were not met and therefore the study is considered to present a possible bias that weakens the reliability of the results and “uncertain bias” (due to lack of information). The Newcastle-Ottawa scale (8) was used to measure the quality of non-randomized observational studies; it was considered “low risk of bias” in the case of a star score > 6 and “high risk of bias” for a score ≤ 6. Case series studies were evaluated using the MOGA scale. The degree of inter-examiner agreement of the methodological quality assessment was obtained with the Cohen kappa test, following the scale proposed by Landis and Koch (9).

- Data synthesis:

With the aim of summarizing and comparing studies, average data on main variables were grouped for each study group. As the average data found in the analyzed studies came from different samples, weighted arithmetic mean was calculated to obtain feasible outcomes. A meta-analysis was not able to be performed due to the lack of randomized studies comparing both procedures.

## Results

**Figure 1:**
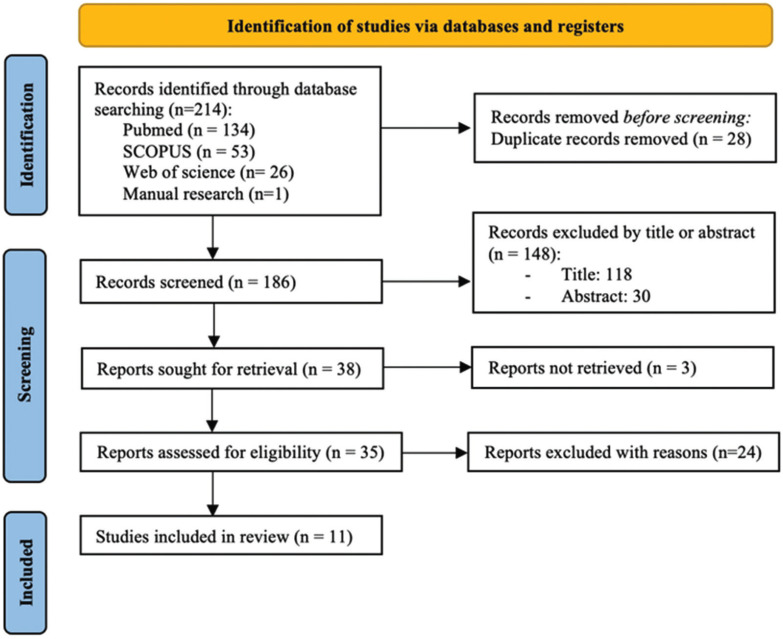
PRISMA flowchart of searching and selection process of titles during systematic review.

- Study selection:

A total of 214 articles were obtained from the initial search process: Medline-PubMed (n=134), SCOPUS (n=53) and the Web of Science (n=26). In addition, 1 title was obtained through manual searching (references list and primary sources). Of these publications, 38 were identified as potentially eligible articles through screening by titles and abstracts. The full-text articles were subsequently obtained and thoroughly evaluated. As a result, 11 articles met the inclusion criteria and were finally included in this systematic review (Fig. 1). The k value for inter-reviewer agreement for study inclusion was 0.87 (titles and abstracts) and 1.0 (full texts) indicating ‘‘good’’ and ‘‘full’’ agreement, respectively, according to the Landis and Koch criteria (9).

- Study characteristics:

Of the 11 articles included in this review, 5 articles described bone augmentation using the Onlay block grafting technique (10-14), 5 the GBR technique (15-19) and 1 performed a comparison of both treatment groups (20). Finally, 5 articles were randomized controlled (10,12,13,16,18), 3 were prospective studies (17,19,20) and 3 were case series (11,14,15). In randomized studies, the patient was the random assignment unit. A total of 167 patients were treated: 94 treated with the GBR technique, 63 using the Onlay block graft technique and 10 patients treated with both techniques. A total of 202 grafted areas were intervened: 117 with grafts according to the GBR technique, 73 according to the Onlay block grafting technique and 12 areas treated with both techniques (Table 1).

**Table 1:**
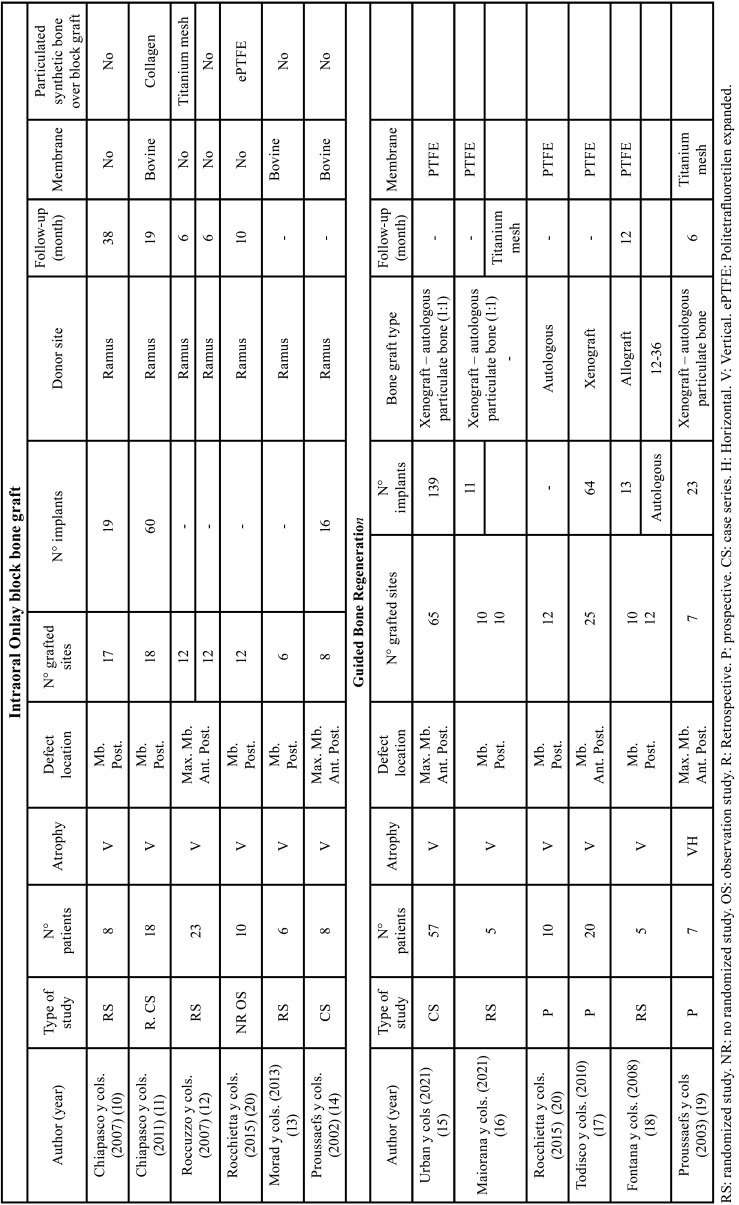
Characteristics of the included studies.

**Figure 2:**
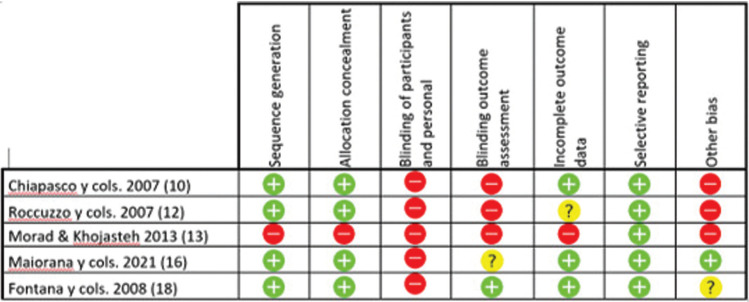
Randomized studies risk of bias following Cochrane’s guidelines.

**Figure 3:**
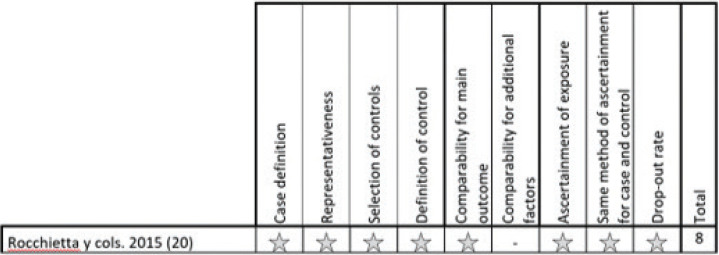
Observational non randomized studies according to Newcastle-Ottawa scale- observational studies with control group non randomized.

**Figure 4:**
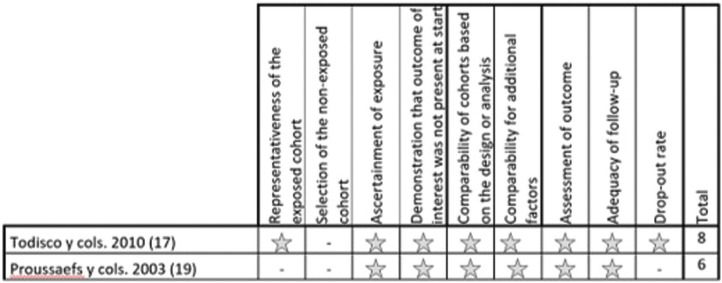
Observational non randomized studies according to Newcastle-Ottawa scale- cohorts observational studies without control group.

- Risk of bias:

For randomized studies, a high risk of bias was considered in all 5 studies (Fig. 2). For non-randomized observational studies, the risk of bias was considered low in 2 studies and high in 1 study (Figs. 3,4). For case series studies, a high risk of bias was considered by the nature of the study type. Detection bias (blinding of staff, patients, and assessors) was the item with the highest risk of bias. The k value (Cohen kappa test) regarding to the agreement between the reviewers of the methodological quality was 0.78 according to the scale of Landis & Koch (9).

- Synthesis of results:

Bone gain and superficial bone resorption:

In relation to Onlay block bone grafts, 5 studies provided data on bone gain (10,12-14,20). Vertical bone gain mean was 4.05 mm, ranged from 2.91 mm (20) to 5,12 mm (14). Superficial resorption rate mean was 17.70%, ranged from 8,5% (20) to 34.5% (12). The highest gains and resorption values were found in studies that did not use membranes; mean values fluctuated between 3.6 mm (12) and 5.12 mm (14) when the blocks were not covered with membranes and, between 2.91 mm (20) and 4.8 mm (12) when they were used. In terms of resorption, when they were not covered with membranes, they ranged from 13 (10) to 34.5% (12) and, when membranes where used, they ranged between 8.5% (20) and 13.5% (12).

Regarding to the GBR technique, 6 studies provided data on bone gain (15–20). Vertical bone gain mean was 4.7 mm, ranged from 1.5 mm (16) to 5.24 mm (17). Superficial resorption rate mean was 15.08%. The highest gains corresponded to the studies that used PTFE membranes (compared to titanium mesh); means values ranged between 4.1 mm (18) and 5.24 mm (17) (Table 2).

**Table 2:**
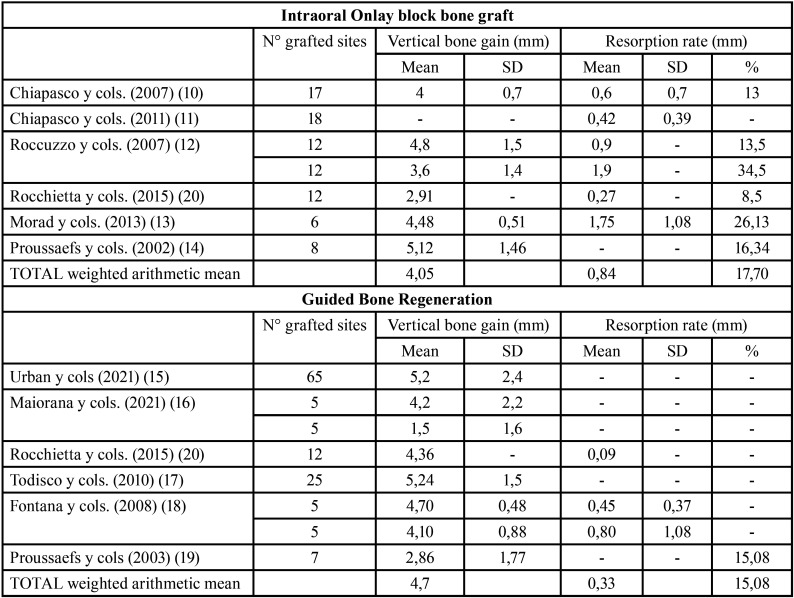
Descriptive outcomes on bone gain and superficial resorption.

- Complications related to bone grafting:

Six studies reported data regarding complications related to Onlay block bone grafting procedure (10–14,20) and 6 to the GBR technique (15-20). In respect of Onlay block grafts, 17 complications were reported in 85 grafted sites versus 15 complications in 129 grafted sites using the GBR technique (20% versus 11.6%, respectively) (Table 3).

**Table 3:**
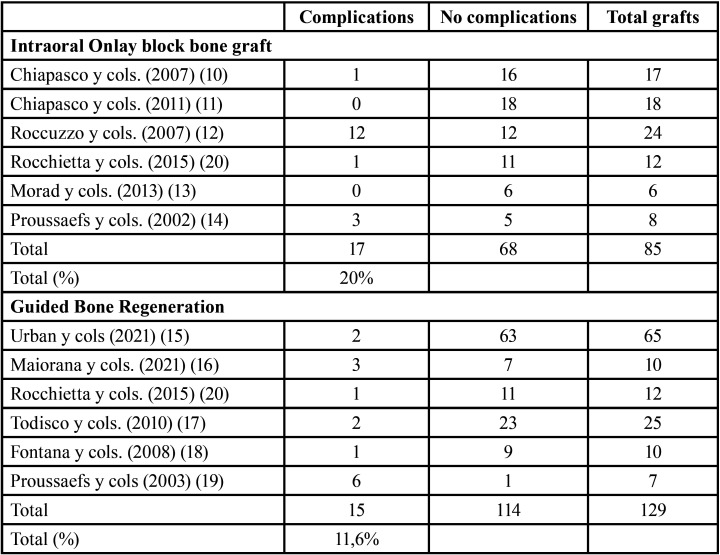
Descriptive outcomes on postoperative complications related to bone grafting procedure.

In the Onlay block group, the most frequent complication of the recipient area was wound dehiscence with graft exposure (n=7) (10,12,14), followed by early membrane exposure (n=4) (12) and/or insufficient graft volume (n=4) (12).

In the GBR technique, the most frequent complication was wound dehiscence with early membrane exposure (n=10) (15-19), following by graft infection (n=3) (15,16,20). Regarding the donor area, paresthesia was the most frequent complication in both groups (n=8 in the block group (10-12); n=3 in the GBR group (18,19)), although in most cases, it was resolved in 1-12 weeks.

- Implant survival and success rates and peri-implant marginal bone loss:

Regarding the Onlay block group, 2 studies evaluated survival and success rates, as well as peri-implant marginal bone loss (10,11). Implant survival and success rates means were 100% and 92.23%, respectively, at 12-48 months post loading, and the peri-implant marginal bone loss mean was 0.22 mm at 12 months post loading.

To the GBR group, 2 studies (17,18) provided data on survival rate, 1 study (17) on peri-implant marginal bone loss, but no study provided data on success rate. The implant survival rate mean was 100% at 12-36 months post loading and the peri-implant marginal bone loss mean was 0.95 mm at 12 months post loading (Table 4).

**Table 4:**
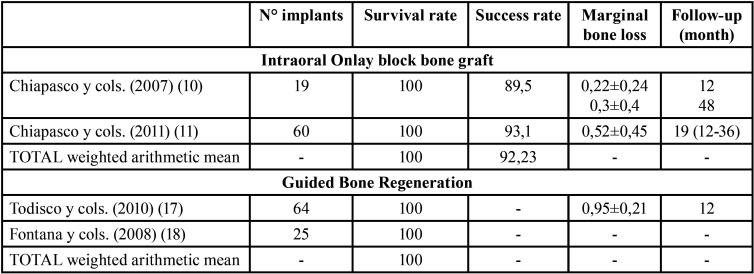
Descriptive outcomes of implant survival and success rate and marginal bone loss.

## Discussion

The lack of randomized studies comparing both techniques made it impossible to perform a meta-analysis. For this reason, the results presented here should be interpreted with caution and were presented descriptively in each study group.

- Bone gain and superficial bone resorption:

The results of this systematic review revealed higher bone gain and lower resorption with GBR in vertical augmentation of the posterior mandibular ridges. These results agree with those reported in other systematic reviews (4,21). Elnayef *et al*. (21) found gain values of 3.83 mm for GBR and 3.47 mm for blocks, and resorption values of 0.90 mm for GBR and 1.21 mm for blocks. These results could be attributed to the fact that the GBR procedure is not conditioned by the availability of bone in the patient’s donor site, as is the case with the bone block grafting, where the block obtained has limited anatomical dimensions. Also, the fact that surface resorption was lower with GBR may be due to the more rapid revascularization that occurs in the particulate graft, as opposed to the block graft, which has a cortical component that has been associated with greater resorption (22).

 Complications related to bone grafting:

The results of the present systematic review revealed a higher complication rate with blocks. These agree with what has been published in other systematic reviews (4,21). Urban *et al*. (4) reported complication values of 26.1% for the blocks and 12.1% for the GBR. However, these results are often controversial since the systematic reviews published by Rocchietta *et al*. (23) and Chiapasco *et al*. (24) revealed a higher complication rate in the GBR group. In reference to the recipient site, the most frequently observed complication in the block group was graft exposure, while in the GBR it was membrane exposure. In reference to the donor site, a higher complication rate was observed in the vertical bone augmentation technique using Onlay blocks, probably related to the graft harvesting process. Indeed, the amount of particulate bone harvested for vertical ridge augmentation is usually less than the amount of bone harvested for intraoral block bone grafting, as the autogenous particulate graft is usually mixed with synthetic bone, thus reducing the subsequent need to harvest a significant amount of autologous bone. Therefore, harvesting the graft by milling the cortico-cancellous block graft results in a greater chance of damaging the nerves and blood vessels near the donor site than harvesting the particulate graft. However, the sensory disturbances found in the GBR studies could be attributed to the preparation of the recipient bed where the release of the flap through the releasing periosteal incisions could injure some mentonian nerve fibers.

- Implant survival and success rates and peri-implant marginal bone loss:

In the literature, many different criteria are used to determine the survival and success rates of dental implants. In addition, there is a discrepancy in the reported results when the main unit of analysis is the patient rather than the dental implant (25). The articles in the present review report high survival rates; this agrees with what has been reported in other systematic reviews (21,25). The systematic review published in 2015 by Aloy-Prósper *et al*. (26) on block grafts concluded that the survival and success rates of implants placed in atrophic alveolar ridges reconstructed with block bone grafts were similar to those of implants placed in native bone. Regarding peri-implant marginal bone loss at 12 months, our results reveal greater bone loss in the GBR technique. This agrees with the systematic review by Keestra *et al*. (25); they reported a mean loss of 0.60 - 1.46 mm around block augmented ridge loaded implants and a mean loss of 1.01 - 1.86 mm in the GBR augmented ridge. However, the results should be interpreted with caution as the value obtained for marginal bone loss at 12 months in the GBR group (0.95mm) was only achieved by the study of Todisco *et al*. Furthermore, Keestra *et al*. (25) concluded that marginal bone loss around implants placed in atrophic alveolar ridges reconstructed vertically with block bone grafts or GBR was similar to that of implants placed in native bone.

Despite the limitations, both techniques offer a predicTable way to reconstruct atrophic mandibular alveolar ridges, although the GBR technique appears to achieve greater bone gain and less surface resorption. However, current evidence is limited due to inadequate follow-up and lack of information on methodological quality.
